# Sensitivity of Honeybee Hygroreceptors to Slow Humidity Changes and Temporal Humidity Variation Detected in High Resolution by Mobile Measurements

**DOI:** 10.1371/journal.pone.0099032

**Published:** 2014-06-05

**Authors:** Harald Tichy, Wolfgang Kallina

**Affiliations:** Department of Neurobiology, Faculty of Life Sciences, University of Vienna, Vienna, Austria; University of Arizona, United States of America

## Abstract

The moist cell and the dry cell on the antenna of the male honeybee were exposed to humidities slowly rising and falling at rates between –1.5%/s and +1.5%/s and at varying amplitudes in the 10 to 90% humidity range. The two cells respond to these slow humidity oscillations with oscillations in impulse frequency which depend not only on instantaneous humidity but also on the rate with which humidity changes. The impulse frequency of each cell was plotted as a function of these two parameters and regression planes were fitted to the data points of single oscillation periods. The regression slopes, which estimate sensitivity, rose with the amplitude of humidity oscillations. During large-amplitude oscillations, moist and dry cell sensitivity for instantaneous humidity and its rate of change was high. During small-amplitude oscillations, their sensitivity for both parameters was low, less exactly reflecting humidity fluctuations. Nothing is known about the spatial and temporal humidity variations a honeybee may encounter when flying through natural environments. Microclimatic parameters (absolute humidity, temperature, wind speed) were measured from an automobile traveling through different landscapes of Lower Austria. Landscape type affected extremes and mean values of humidity. Differences between peaks and troughs of humidity fluctuations were generally smaller in open grassy fields or deciduous forests than in edge habitats or forest openings. Overall, fluctuation amplitudes were small. In this part of the stimulus range, hygroreceptor sensitivity is not optimal for encoding instantaneous humidity and the rate of humidity change. It seems that honeybee's hygroreceptors are specialized for detecting large-amplitude fluctuations that are relevant for a specific behavior, namely, maintaining a sufficiently stable state of water balance. The results suggest that optimal sensitivity of both hygroreceptors is shaped not only by humidity oscillation amplitudes but also according to their impact on behavior.

## Introduction

Hygroreceptors provide an insect with background information such as the direction and rate with which atmospheric humidity is changing. This information contributes significantly to the insect's ability to maintain a stable water balance by avoiding dangerous and preferring favorable humidities. Electrophysiological identification of hygroreceptors has been successfully accomplished in only few species [1], [2]. From their responses to changes in humidity, hygroreceptors can be divided into two antagonistic categories: moist cells which respond with a sharp rise in the rate of discharge to rising humidity, and dry cells which respond with a sharp rise in discharge rate to falling humidity. Moist and dry cells have been found always together in the same sensillum on the insect antennae. Thus, both types of cells have identical receptive fields which could well enhance the detection of local changes in humidity.

In earlier studies, humidity changes were produced by switching rapidly from one conditioning air stream at a constant humidity to a second at a different constant humidity [3]–[7]. The advantage is that humidity transients, if sufficiently large, cause significant changes in the ongoing discharge rates, illustrating the antagonistic members of the two hygroreceptors. The disadvantage, however, is that the humidity of the stimulating second air stream cannot be assigned directly to the hygroreceptive sensillum during a rapid humidity change. It is only possible to assign instantaneous humidity values to the hygroreceptive sensillum or even to structures involved in humidity transduction when humidity changes slowly so that the sensillum's moisture content is in equilibrium with ambient humidity. Therefore, a second method of humidity stimulation was developed that utilized slow and continuous changes in humidity. Here, the rates are low enough so that the difference between the sensillum's moisture content and the humidity of the stimulating air stream is insignificant and thus permits air humidity to stand for that of the sensillum. Furthermore, when the humidity moves slowly and continuously up and down at different rates, the discharge rates of the moist and dry cells reflect – from instant to instant – not only a succession of humidities but also the rate of change [8]–[10]. Thus, the moist cell and the dry cell signal the actual humidity along with the direction and speed of a humidity change.

Understanding the function of hygroreceptors requires knowledge of the adequate stimulus. The relative humidity is obviously the most familiar and practical way of measuring atmospheric humidity and defining the content of water of hygroscopic materials. The water content of a human or horse hair under tension changes its length due to changes in the relative humidity of the air. Accordingly, it was assumed that the sensillum wall acts as a hygro-mechanical transducer which changes its dimension due to the uptake (adsorption) and loss (desorption) of water, much like a hair hygrometer depending on changes in relative humidity [3]–[7]. Relative humidity, however, is a measure of the actual amount of water vapor in the air as a percentage of the saturated water vapor pressure at that temperature. As such it indicates the humidity gradient which promotes the evaporation of water from a sensillum surface at a single constant temperature. Relative humidity becomes irrelevant if observations are extended over a range of temperatures because it is not a direct measure of any absolute quantity of water vapor but merely a ratio between two known values. The capacity of air to hold water vapor increases rapidly with temperature, approximately doubling every 10°C. Thus, the same relative humidity can indicate very different atmospheric moisture conditions. In other words, two different regions having the same relative humidity do not imply similar atmospheric water conditions unless temperatures are also identical. Under these circumstances the saturation deficit is the exact measure; it is the difference between saturated vapor pressure and actual vapor pressure at a given temperature, thus indicating the vapor pressure gradient between the sensillum surface and its environment at any temperature. In a recent study we demonstrated that the responses of the cockroach's hygroreceptors to slow and continuous changes in saturation deficit correspond – at different constant temperatures – to a psychrometer rather than a mechanical hygrometer [9]. Since a psychrometer measures the humidity (or the dryness) of the air by means of evaporative temperature depression, cooling the sensillum surface will lead one to assume equal temperature decrease of the sensory cell soma including the spike generating region. However, the amount of moisture on the sensillum surface for adequate evaporation will certainly be very small so that cooling of the sensillum surface will lower the temperature of the dendrites which project into the external sensillum structure but not that of the cell soma which are tightly encased by sheath cells and embedded in the antennal epithelial cells.

Regardless of how the process of humidity transduction is best described, the most important function of insect hygroreceptors is to acquire information about changes in atmospheric humidity. Hygroreceptors represent two input parameters (instantaneous humidity and its rate of change) with a single output parameter (impulse frequency) [8]–[10]. However, information maximization is not enough. It would not make sense to pack a lot of information into the output that is of little relevance for the insect. Hygroreceptors may not seek an efficient representation of the whole humidity scale from extremely dry to extremely moist; rather, their optimization may be heavily biased towards those humidity stimuli that are most relevant for behavioral constrains, such as regulating body water content. This raises the question of what attributes of the humidity stimulus occurring in an insect's natural environment are encoded best by the moist and dry cells. In order to investigate if and how hygroreceptors are optimized with respect to their natural environment, we here perform a systematic study with oscillating humidity changes. By examining a set of three oscillation amplitudes (roughly 50%, 30% and 10% relative humidity) that cover different parts of the gross humidity range (0–100% relative humidity) it should be possible to determine the properties of the humidity stimulus which are most efficiently represented in the hygroreceptors activity.

**Figure 1:**
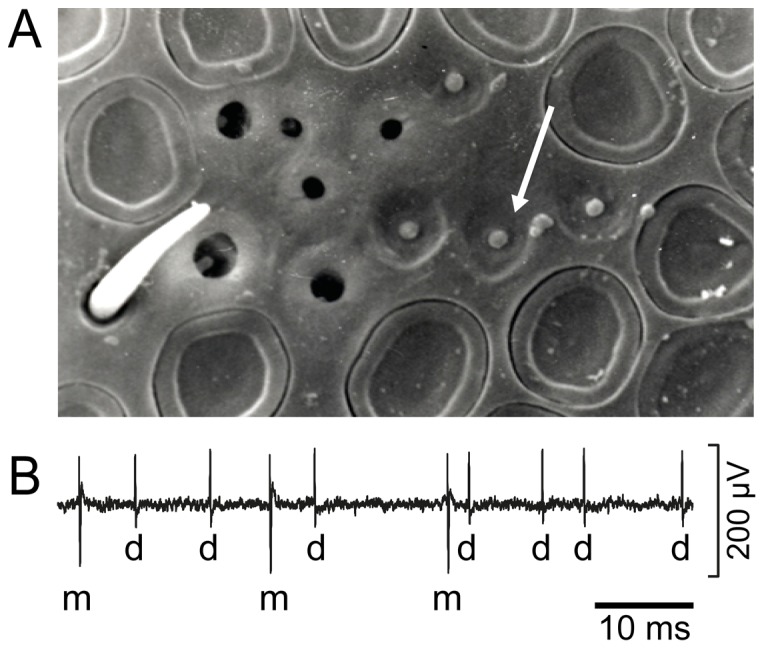
Scanning electron micrograph of hygroreceptive sensilla on male honeybee antenna and extracellular recorded activity of moist and dry cell. **A.** The cap of the sensillum (*arrow*) is visible in the central opening of a swallow cuticular depression of the antennal surface. **B.** Large amplitude impulses are produced by moist cell (*m*) and small amplitude impulses by dry cell (*d*). Amplitude differences are fairly typical.

We chose to study the dynamic sensitivity of the moist and dry cells in the male honeybee. The moist and dry cells of the honeybee offer advantages. They occur together in peg-shaped sensilla located in heavily walled pits which are readily found on worker bee and drone antennae (Fig. 1A) and some data are already available [11]–[13]. However, it was technically difficult to simultaneously record the activity of the moist and dry cell with the same electrode. The best long-term recordings were obtained from drones. Their hygroreceptors withstood several series of quantitative stimuli, which ensures that their sensitivity to a wide range of humidity stimuli can be determined while preserving their weak and irregular responses to low-amplitude humidity changes. It should therefore be accessible to the question of what information about fluctuating humidity changes is represented in the activity of the moist and dry cells.

Our knowledge of the properties of humidity stimuli occurring in the insect's natural environment is limited to stationary measurements. Nothing is known about the amplitudes and rates of humidity changes a flying insect may encounter in its natural environment. Plausible ranges of humidity fluctuations could be measured from an automobile traveling with constant speed through different landscapes. Such measurements would indicate what temporal humidity profiles may hit the hygroreceptors of a flying insect. Normal worker bee flight speed is about 7.1 to 8.2 m/s (25.5 to 29.5 km/h [14]), when seeking a food source. Of course, drones are not engaged in exploiting sources of pollen, nectar or water. Since they do not mate with the virgin queen of the same hive, they fly from hive to hive, arrive at the drone congregation area and mate in flight. Drones may fly at lower speed than worker bees. Therefore, they will be confronted with lower rates of humidity change. If flying speed is known, the rates of humidity change can be calculated from the data of the mobile measurements.

## Material and Methods

### Electrophysiological recordings

The drones of the honey bee, *Apis mellifera*, were obtained from a local beekeeper. An animal was fixed to a holder with adhesive tape, and one antenna was attached to a narrow support with dental cement (Harvard Cement) for unobstructed humidity stimulation. The hygro-thermoreceptive sensilla (coelocapitular sensilla [11]–[13]) are grouped in a shallow depression on the mid-region of each antennal segment. Identification was based on the responses to humidity stimulation. Action potentials were recorded between two electrolytically sharpened tungsten wires, one inserted at an angle of about 45° into the sensillum base, and the other lengthwise about 2 mm into the tip of the antenna. After amplification, the band pass (0.1–3 kHz) filtered signals were passed through an AD-converter (1401 plus, Cambridge Electronic Design; 12-bit; 10 kHz) and fed into a PC for online recording. The digitized impulses and the voltage output of the electronic flow meters were displayed online on a monitor, stored on a hard disk and sorted off-line using Spike2 software (Cambridge Electronic Design, UK). Spike parameters were extracted from the stored waveform channel and sampled to form templates. Detected spikes were then subjected to the template-matching system to create or modify the templates. Each spike was compared against the templates and, each time a template was confirmed, it was added to the template by overdrawing. Adding a spike to a template may change the shape and width of the template outlines. Thus the template boundaries display homogeneity of classification.

### Response evaluation

Impulse frequency (*F*, impulses/s) was calculated from running averages of three consecutive 0.5-s intervals.

### Control of humidity and temperature in the electrophysiological experiments

Air from a pressure-regulated source was cleaned, dried and split into two streams. Their flow rates were equalized by matching the rates in mass flow meters. The first stream was bubbled out through many openings in a polyethylene tube firmly anchored in a tank containing ion-exchange purified water at constant depth and at 42°C. The second stream was conducted through the tank in a spiral tube and remained dry as it was warmed to 42°C. The temperature of the two streams was then set at different temperature levels by driving them through a further self-made, thermostatically controlled heat exchanger. After emerging from the heat exchanger, the two air streams passed through electrical proportional valves (KWS 3/4, Kolvenbach) and then were combined to a single stream. The water vapor pressure of this stream was sinusoidally modulated by mixing the two streams in a ratio determined by the proportional valves. To hold the flow rate of the mixed air constant at 2.5 m/s, the control voltages (AD-converter, 1401 plus, Cambridge Electronic Design) of the proportional valves were phase shifted by 180°. The mixed air was divided into two streams. For stimulation, the first stream was directed towards the antenna by way of a Plexiglas tube 7 mm in diameter. The hygroreceptive sensillum was 5 mm away from the outlet of the tube. The temperature within the air stream was measured within ±0.03°C by a small thermistor (250×400 µm; Fenwall Electronics, BC 32 L1) 3 mm downstream from the sensillum. By passing the second air stream through a 1-cm^3^ detection chamber of an UV-absorption hygrometer (K 20, Campbell Scientific), water vapor density was measured at a rate of 100 Hz. The voltage outputs of the hygrometer and the thermistor were passed through the AD-converter (1401 plus, Cambridge Electronic Design), fed into the PC and recorded online. Based on the digitized signals of the hygrometer and the thermistor, the water vapor pressure (*Pw*) and the relative humidity (*rH*) were monitored offline. The saturation water vapor (*Ps*) and the saturation deficit (*SD*) were determined offline by running a self-written script.

### Mobile measurements

Air humidity was measured by an UV krypton absorption hygrometer at a rate of 100 Hz (K 20, Campbell Scientific), ambient temperature in real time by a fine-wire thermocouple (wire diameter 13 µm, Type E, Campbell Scientific), and wind speed in real time by a hot wire anemometer. These sensors were installed at the front end of a 4-m rod that was mounted with its back end on the leading edge of the roof rack of an automobile so that it juts out on the right side. In this way, data were collected at a height of 1.5 m above the ground and more than 3 m within the roadside vegetation. The safety distance of more than 5 m minimized the influence of the exhaust fumes. The voltage outputs of the sensors were displayed on an oscilloscope inside the automobile and recorded on a DAT-recorder. In the laboratory, the data were passed through a CED 1401-micro (Cambridge Electronic Design, 12 bit, 300 Hz) interface, stored on a hard disc of a PC and analyzed using the commercial software Spike2.

Measurement trips lasting 1.5 to 2 h were made in the countryside of Lower Austria (Tullnerfeld, Marchfeld, Schneebergland) on calm days in June and July during late morning and early afternoon. The sky was generally clear, with some scattered clouds. The automobile travelled at a mean speed of 25 km/h (7 m/s) along vegetated roadsides, open farm fields, grasslands with clusters of trees shadowing the ground, and edge habitats. Deciduous forests with low canopy cover were also crossed. The trips were continuous with no stops or road intersections. No specific permissions were required for the measurement trips in these landscapes. The field studies did not involve endangered or protected species.

**Figure 2:**
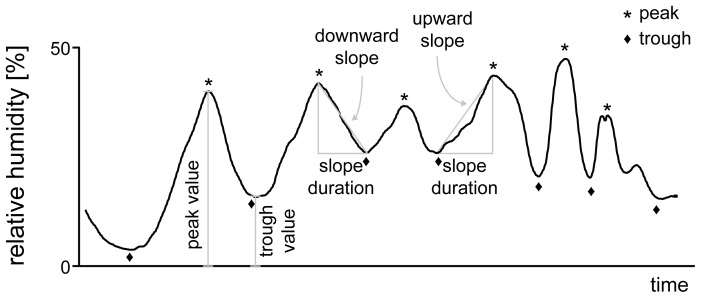
Humidity profile showing the parameters analysed. Peak and trough amplitudes are values over the zero level, durations of the upward and downward slopes are the periods between successive peaks and troughs, and the values of the upward and downward slopes are the velocities with which humidity is rising and falling, respectively.

The basic measures chosen to characterize the temporal profiles of temperature, humidity and wind speed were the amplitudes and positions of the peaks and troughs, and the values of the rising and falling slopes (Fig. 2). Peaks and troughs were subjected to an amplitude matching system that defines how much a signal must rise before a peak and fall after it (to detect a peak) or how much a signal must fall before a trough and rise after it (to detect a trough). These values were 3% for the relative-humidity trace, 2 mbar for the saturation-deficit trace, 0.5 m/s for the wind-speed trace, and 0.3°C for the temperature trace. Peaks and troughs were rejected if the minimum fall after a peak was <3% relative humidity (2 mbar, 0.5 m/s and 0.3°C, respectively) of the rise before the peak, or if the minimum rise after a trough was <3% relative humidity (2 mbar, 0.5 m/s and 0.3°C, respectively) of the fall before the trough. The characteristic values of each temporal profile were then averaged for statistical measures over the whole recording period.

## Results

### Responses of the hygroreceptors

**Figure 3:**
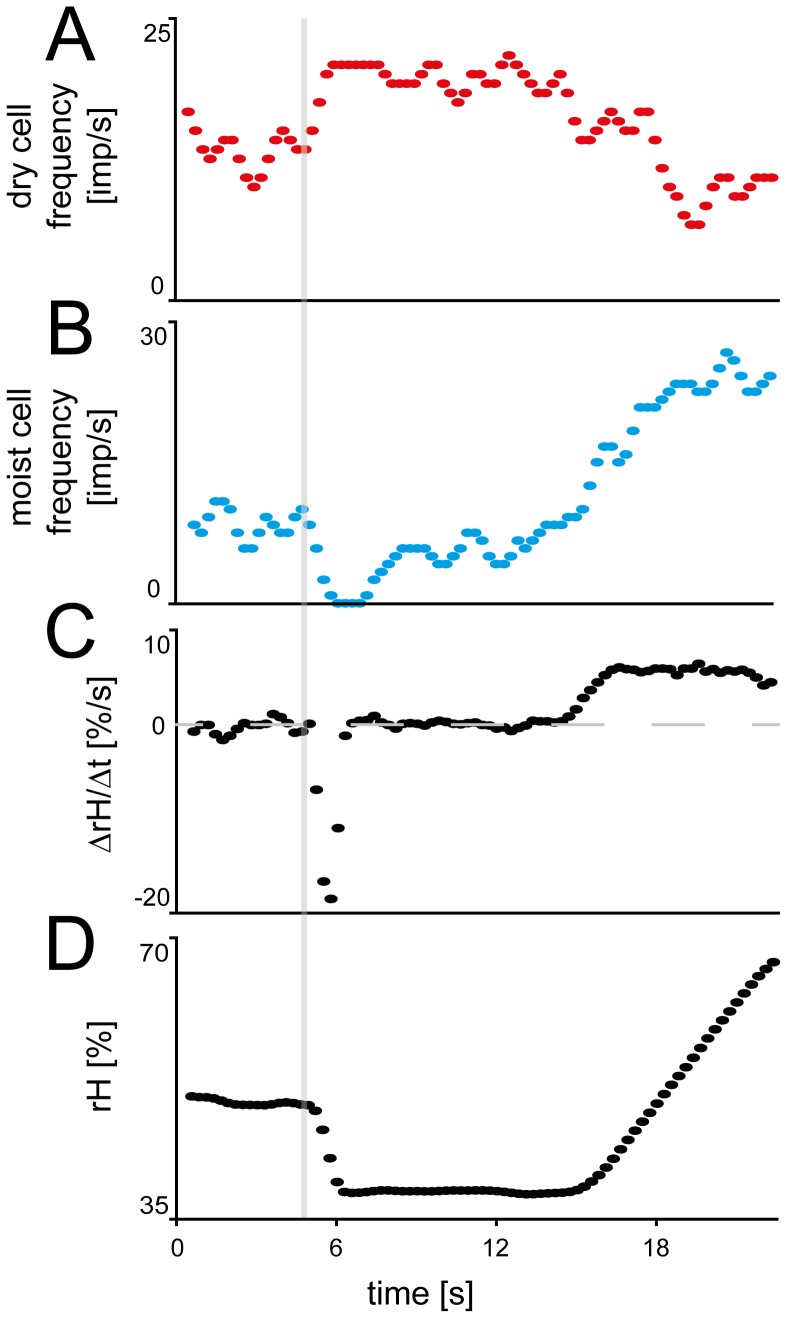
Simultaneously recorded responses of a pair of moist and dry cells from a single sensillum to transient and ramp-like changes in humidity. A and B. Time course of the instantaneous impulse frequency of the moist cell and the dry cell, respectively. Bin width, 1*rH* relative humidity.

In both worker bees and drones, the hygroreceptive sensilla are located at the mid region of most antennal segments. Their external structure is shaped like a mushroom with a slightly dilated cap. They are positioned in the central opening of shallow cuticular depressions so that only the cap is visible from outside (Fig. 1*A*). An electrode inserted into the shallow cuticular depression near the edge of the wall revealed the activity of the moist cell and dry cell, distinguishable by the amplitude and form of their impulses (Fig. 1*B*). The two types of hygroreceptors were identified by their responses to humidity changes of an air stream flowing over the antenna. Rising humidity increased the impulse frequency of the moist cell and decreased that of the dry cell, and, conversely, falling humidity increased the impulse frequency of the dry cell and decreased that of the moist cell (Fig. 3*A*–*D*).

**Figure 4:**
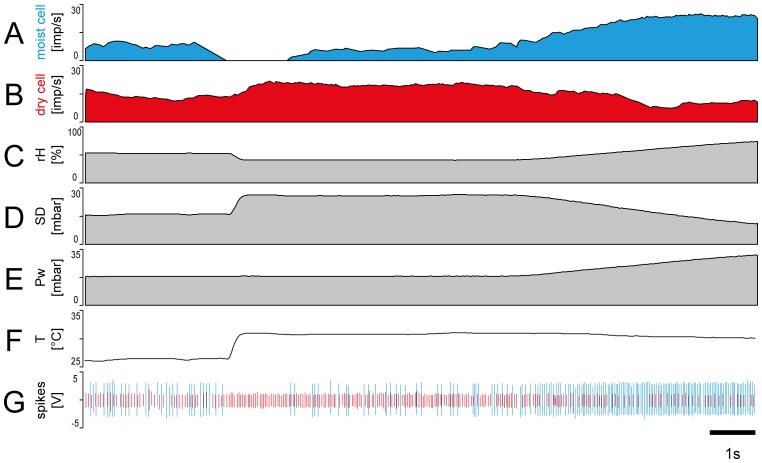
Simultaneously recorded responses of a pair of moist and dry cells from a single sensillum to transient and ramp-like changes in humidity. **A.** and **B.** Antagonistic responses of moist and dry cells to humidity changes produced by an increase in temperature at constant water vapor pressure or an increase in water vapor pressure at constant temperature. Instantaneous impulse frequency; bin width, 1 s. **C.** Time course of relative humidity. Final 3 seconds of a 20 s presentation of an air stream with 49.5% *rH*, followed by a humidity drop to 37.5% *rH* which was held for 7 s; stimulus direction was then reversed by presenting a ramp-like humidity increase from 41.0% to 74.1% *rH*. **D.** Time course of saturation deficit. The drop in relative humidity shown in C is expressed as jump in saturation deficit from 15.9 mbar to 25.0 mbar, and the ramp as a slow decrease from 25.0 to 10.6 mbar. E. Time course of water vapor pressure. The humidity ramp was produced by changing the water vapor pressure slowly from 18.1 to 31.5 mbar. F. Time course of temperature. Rapid humidity change produced by a temperature drop from 26°C to 30°C. G. Digitized action potentials of the moist and dry cells recorded simultaneously with a single electrode and discriminated on-line. *Pw* water vapor pressure, *rH* relative humidity, *SD* saturation deficit, *T* temperature, *V* volt.

The same antagonistic responses of the two cell types to an increase or decrease in the relative humidity can also be elicited by changing the air temperature. For example, a sudden increase by 4.5°C from 26.4 to 30.9°C (Fig. 4*F*) at a constant absolute humidity of 18.1 mbar (Fig. 4*E*) resulted in a 12% decrease in the relative humidity (Fig. 4*C*) and a 9 mbar increase in the saturation deficit (Fig. 4*D*). This caused the moist cell to interrupt its discharge briefly (Fig. 4*A,G*) and the dry cell to sharply raise impulse frequency (Fig. 4*B,G*). Both cell types also respond antagonistically to a slow, ramp-like increase in the water vapor pressure. For example, when at a constant temperature of 30.9°C the absolute humidity was slowly increased by 13.4 mbar from 18.1 to 31.5 mbar (Fig. 4*E*; last 5 s of the recording), then the relative humidity slowly increased by 33% (Fig. 4*C*) and the saturation deficit slowly decreased by 14.4 mbar (Fig. 4*D*). This caused the discharge rate of the moist cell to continuously increase (Fig. 4*A,G*) and that of the dry cell to continuously decrease (Fig. 4*B,G*).

**Figure 5:**
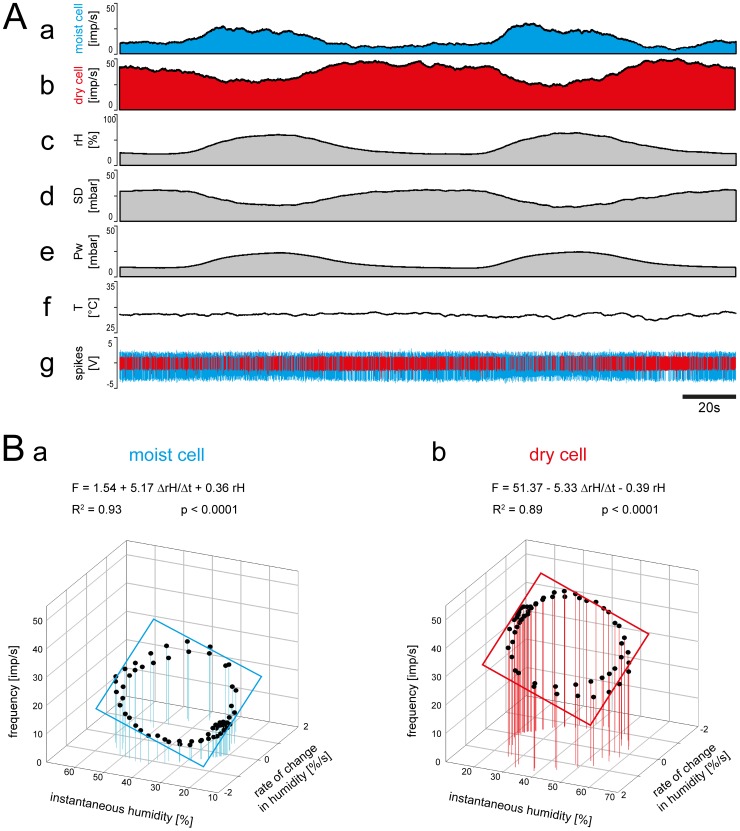
Simultaneously recorded responses of a pair of moist and dry cells from a single sensillum to high amplitude changes in humidity. A. Time course of the responses and the parameters controlled during the recording, and the corresponding activity of the moist and dry cells. *a* and *b*, instantaneous impulse frequency of both cells. *c*, time course of the relative humidity. *d*, time course of the saturation deficit. *e*, time course of the water vapor pressure. *f*, time course of the temperature. *g*, digitized action potentials of the moist and dry cells recorded with a single electrode and discriminated on-line. B. Impulse frequency of the moist and dry cells during the oscillations in relative humidity shown in A plotted as a function of instantaneous relative humidity and its rate of change. Multiple regressions which utilize 3-dimensional planes (*F* = *a*+*b drH/dt*+*c rH*; where *F* is the impulse frequency, and *a* the height of the regression plane) were calculated to determine the simultaneous effects of the rate of change in the relative humidity (*b* slope) and the instantaneous relative humidity (*c* slope) and the response frequency of both cell types. *Pw* water vapor pressure, *R^2^* coefficient of determination, *rH* relative humidity, *SD* saturation deficit, *T* temperature, *V* volt.

Three kinds of experiments were performed involving slowly changing humidity. In the first kind a humidity amplitude of roughly 50% *rH* between 20 and 70% *rH* was covered and the rates of humidity change were between –2% *rH*/s and +2% *rH*/s. In the moist cell, impulse frequency varied between values of 5 and 30 imp/s (Fig. 5*A* *a,g*), and in the dry cell between 22 and 50 imp/s (Fig. 5*A* *b,g*). Impulse frequency of the moist cell tended to be higher at the higher values of instantaneous humidity and lower at the lower values (Fig. 5*A* *a,c*). Conversely, impulse frequency of the dry cell tended to be higher at the lower values of instantaneous humidity and lower at the higher values (Fig. 5*A* *b,c*). Frequency values during humidity oscillations may simply be interpreted as the response to instantaneous humidity, i.e., the succession of humidities at particular instants in time. Impulse frequency, however, is not in step with instantaneous humidity but ahead of it. A second stimulus parameter that is also in advance of instantaneous humidity must influence their responses. The rate of humidity change was the obvious candidate. To estimate the double dependence on instantaneous humidity and its rate of humidity change, the impulse frequency of the moist and dry cells was plotted in Figs. 5 *Ba* and *Bb* as a function of both parameters.

Multiple regressions (*F = a+b drH/dt+c rH*; where *F* is the impulse frequency and *a* the height of the regression plane) were calculated to determine the simultaneous effects of the rate of humidity change (*b* slope) and the instantaneous relative humidity (*c* slope) on the response frequency of both cell types (Fig. 5*B* *a,b*). The slopes demonstrate the two properties that characterize each cell: the sign of the slopes is positive for the moist cell and negative for the dry cell; that is, an increase in both instantaneous humidity and its rate of change raises the impulse frequency of the moist cell and lowers that of the dry cell; and the slopes are similar for the moist and the dry cells, sign ignored. Accordingly, given changes in instantaneous humidity or in the rate of humidity change have similar effects on the frequency of the moist and the dry cell with due consideration of the sign.

The mean values of the instantaneous relative humidity and the rate of humidity change from 12 moist and dry cells are listed in Table 1*A*. The coefficients of determination (*R^2^*) show a strong relationship between impulse frequency, instantaneous humidity and rate of humidity change. For the moist cell, *R^2^* indicates that in a series of oscillating humidity changes, an average of 97% of the variation in impulse frequency can be explained by a multiple regression; for the cold cell, the value is 96%. The orderly relationships of impulse frequency to the instantaneous humidity and its rate of change in both cells provide an opportunity to determine their differential sensitivity. The differential-sensitivity values are the expression of the mean change in impulse frequency for each unit change in stimulus magnitude. On average, differential sensitivity to instantaneous humidity was +0.3 imp/s per % *rH* for the moist cell and – 0.4 imp/s per % *rH* for the dry cell; differential sensitivity to the rate of humidity change was +5.2 imp/s per % *rH*/s for the former and –5.4 imp/s per % *rH*/s for the latter. Thus impulse frequency of both cells can be influenced more by changing the rate of humidity change by 1% *rH*/s than by changing instantaneous humidity by one additional percent. Actual measurements show that an increase of 1 imp/s in the moist cell can be elicited either by a +3.3% *rH* increase in instantaneous humidity (at constant rate of change), or by a +0.19% *rH*/s rate of humidity change. In the dry cell, it takes a decrease of –2.5% *rH* in instantaneous humidity – or a rate of change of –0.18% *rH*/s – to increase frequency by 1 imp/s. Both cells display similar response properties, with due consideration of sign: both are more sensitive to the rate of humidity change than to the humidity at which the change takes place.

**Table 1:** Summary of data used to determine differential sensitivities of the moist and dry cells.

Type of unit	moist cell	dry cell
**A. Large amplitude humidity oscillations**		
Amplitude of humidity oscillations (%rH)	40–50	40–50
Range of the rate of humidity change (%rH s^−1^)	−2 to +2	−2 to +2
Units tested extensively	34	34
Units used for multiple regressions	8	8
Number of multiple regressions	40	40
Number of points/multiple regression	<60	<60
Mean correlation of determination (R^2^)	0.97±0.04	0.96±0.05
Mean differential sensitivity for instantaneous humidity (imp s^−1^/%rH)	+0.3±0.3	−0.4±0.4
Increment of instantaneous humidity (%rH) which results in an increment of 1 imp s^−1^	+3.3	−2.5
Mean differential sensitivity for the rate of humidity change (imp s^−1^/%rH s^−1^)	+5.2±1.0	−5.4±0.9
Increment of the rate of humidity change (%rH s^−1^) which results in an increment of 1 imp s^−1^	+0.19	−0.18
**B. Medium amplitude humidity oscillations**		
Amplitude of humidity oscillations (%rH)	25–30	25–30
Range of the rate of humidity change (%rH s^−1^)	−1.5 to +1.5	−1.5 to +1.5
Units tested extensively	34	34
Units used for multiple regressions	8	8
Number of multiple regressions	40	40
Number of points/multiple regression	<60	<60
Mean correlation of determination (R^2^)	0.76±0.04	0.88±0.05
Mean differential sensitivity for instantaneous humidity (imp s^−1^/%rH)	+0.2±0.08	−0.2±0.06
Increment of instantaneous humidity (%rH) which results in an increment of 1 imp s^−1^	+5.0	−5.0
Mean differential sensitivity for the rate of humidity change (imp s^−1^/%rH s^−1^)	+0.5±0.06	−0.8±0.06
Increment of the rate of humidity change (%rH s^−1^) which results in an increment of 1 imp s^−1^	+2.0	−1.25
**C. Small amplitude humidity oscillations**		
Amplitude of humidity oscillations (%rH)	10–15	10–15
Range of the rate of humidity change (%rH s^−1^)	−1.5 to +1.5	−.5 to +1.5
Units tested extensively	34	34
Units used for multiple regressions	12	12
Number of multiple regressions	40	40
Number of points/multiple regression	>60	>60
Mean correlation of determination (R^2^)	0.12±0.08	0.14±0.09
Mean differential sensitivity for instantaneous humidity (imp s^−1^/%rH)	+0.05±0.06	−0.04±0.05
Increment of instantaneous humidity (%rH) which results in an increment of 1 imp s^−1^	+20	−25
Mean differential sensitivity for the rate of humidity change (imp s^−1^/%rH s^−1^)	+0.4±0.08	−0.6±0.08
Increment of the rate of humidity change (%rH s^−1^) which results in an increment of 1 imp s^−1^	+2.5	−1.6

Values implying variation are means ± standard deviation.

**Figure 6:**
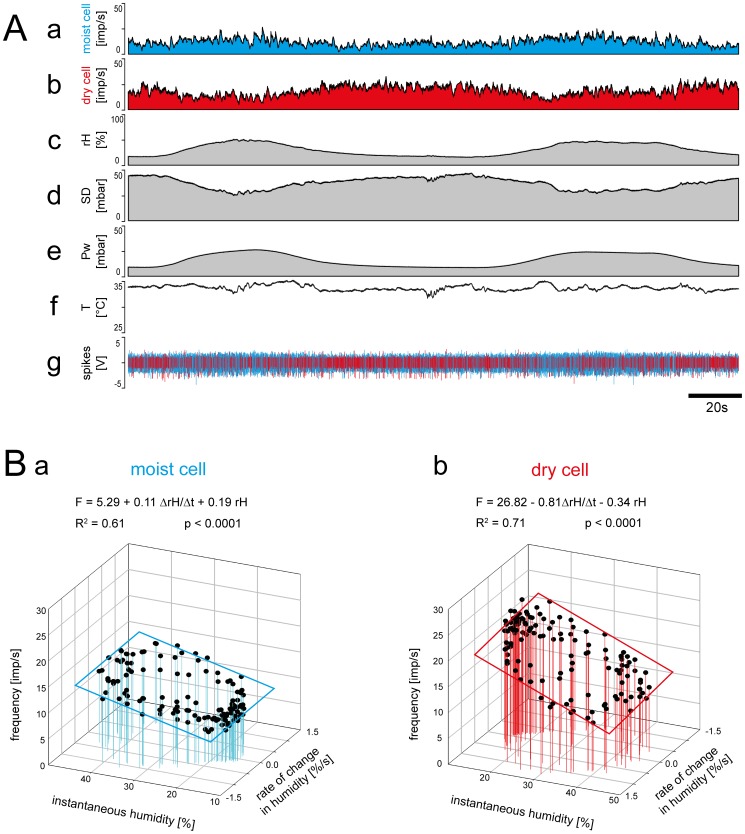
Simultaneously recorded responses of a pair of moist and dry cells from a single sensillum to medium amplitude changes in humidity. **A.** Time course of the responses and the parameters controlled during the recording, and the corresponding activity of the moist and dry cells. *a* and *b*, instantaneous impulse frequency of both cells. *c*, time course of the relative humidity. *d*, time course of the saturation deficit. *e*, time course of the water vapor pressure. *f*, time course of the temperature. *g*, digitized action potentials of the moist and dry cells recorded with a single electrode and discriminated on-line. **B.** Impulse frequency of the moist and dry cells during the oscillations in relative humidity shown in A plotted as a function of instantaneous relative humidity and its rate of change. Multiple regressions which utilize 3-dimensional planes (*F* = *a*+*b drH/dt*+*c rH*; where *F* is the impulse frequency, and *a* the height of the regression plane) were calculated to determine the simultaneous effects of the rate of change in the relative humidity (*b* slope) and the instantaneous relative humidity (*c* slope) and the response frequency of both cell types. *Pw* water vapor pressure, *R^2^* coefficient of determination, *rH* relative humidity, *SD* saturation deficit, *T* temperature; *V* volt.

In the second experiment, slow humidity changes covering a range of roughly 30% *rH* between 15 and 45% *rH* were produced. The rate of humidity change lay between –1.5% *rH*/s and +1.5% *rH*/s (Tab. 1*B*). Moist cell impulse frequency varied between 5 and 20 imp/s (Fig. 6*A* *a,g*), that of the dry-cell between 5 and 30 imp/s (Fig. 6*A* *b,g*). The time course of impulse frequency of both cell types displayed small irregularities. In the moist cell, the higher impulse frequency values were associated with higher humidity (Fig. 6*A* *a,g*), and in the dry cell with the lower humidity (Fig. 6*A* *b,g*). In order to determine the extent to which the impulse frequencies are governed by instantaneous humidity and the rate of humidity change, the frequency values were plotted as functions of both humidity parameters (Fig. 6*B* *a,b*). The emerging coefficients of determination (*R^2^*) indicate that, in a series of oscillating humidity changes, an average of 76% of the variation in the moist-cell' impulse frequency can be explained by a multiple regression; in the dry cell the value is 88%. Thus the mean *b* slope of +0.5 imp/s per % *rH/*s for the moist cell and – 0.8 imp/s per % *rH*/s for the dry cell drawn from all multiple regression provides an estimate for their sensitivity to the rate of humidity change. The mean *c* slope of +0.2 imp/s per % *rH* for the moist cell and –0.2 imp/s per % *rH* for the dry cell estimates their sensitivity to instantaneous humidity. To produce an average increase of 1 imp/s in the moist cell, instantaneous humidity must increase by +5% *rH* or the rate of change by +2% *rH*/s. The corresponding dry cell values are either a decrease in instantaneous humidity of –5% *rH* or a decrease in the rate of change of –1.2% *rH*/s. Thus the dry cell is more sensitive to both humidity parameters.

**Figure 7:**
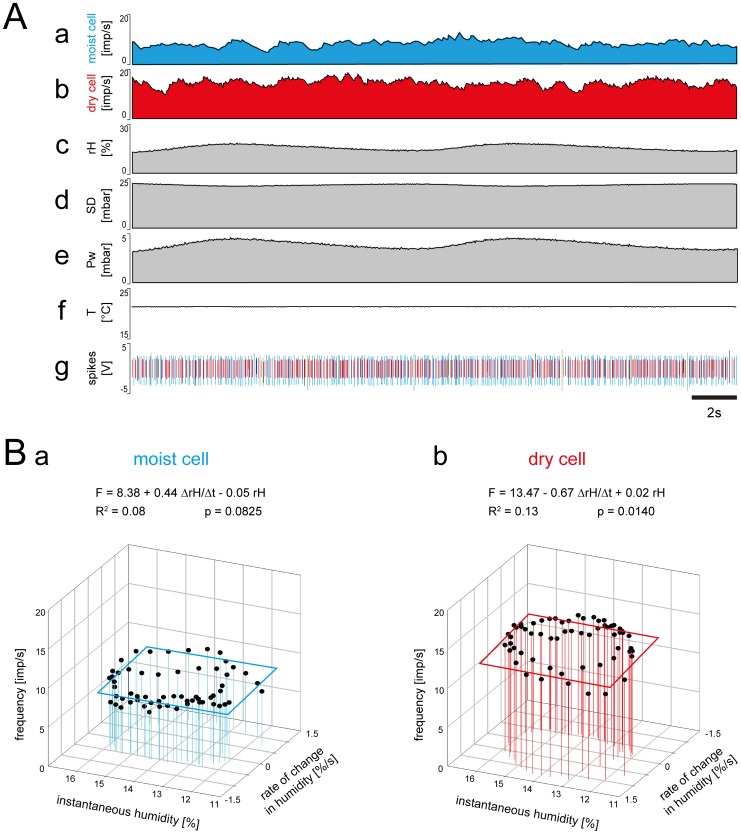
Simultaneously recorded responses of a pair of moist and dry cells from a single sensillum to small amplitude changes in humidity. **A.** Time course of the responses and the parameters controlled during the recording, and the corresponding activity of the moist and dry cells. *a* and *b*, instantaneous impulse frequency of both cells. *c*, time course of the relative humidity. *d*, time course of the saturation deficit. *e*, time course of the water vapor pressure. *f*, time course of the temperature. *g*, digitized action potentials of the moist and dry cells recorded with a single electrode and discriminated on-line. **B.** Impulse frequency of the moist and dry cells during the oscillations in relative humidity shown in A plotted as a function of instantaneous relative humidity and its rate of change. Multiple regressions which utilize 3-dimensional planes (*F* = *a*+*b drH/dt*+*c rH*; where *F* is the impulse frequency, and *a* the height of the regression plane) were calculated to determine the simultaneous effects of the rate of change in the relative humidity (*b* slope) and the instantaneous relative humidity (*c* slope) and the response frequency of both cell types. *Pw* water vapor pressure, *R^2^* coefficient of determination, *rH* relative humidity, *SD* saturation deficit, *T* temperature, *V* volt.

In the third experiment, the humidity changes covered roughly 10% *rH* between 10 and 20% *rH*. The rate of humidity change was between –1.5% *rH*/s and +1.5% *rH*/s (Tab. 1*C*). In the moist cell, impulse frequency varied from 5 to 10 imp/s (Fig. 7*A* *a,g*), in the dry cell from 5 to 15 imp/s (Fig. 7*A* *b,g*). Plotting the impulse frequency of both cells as a function of instantaneous humidity and its rate of change yielded flat regression planes (Fig. 7*B* *a,b*). For the moist cell, *R^2^* indicates that, in a series of oscillating humidity changes, an average of only 12% of the variation in impulse frequency can be explained by a multiple regression; for the dry cell, the value was 14%. Thus, both cells are only slightly affected by humidity changes, if at all.

### Mobile measurements

**Figure 8:**
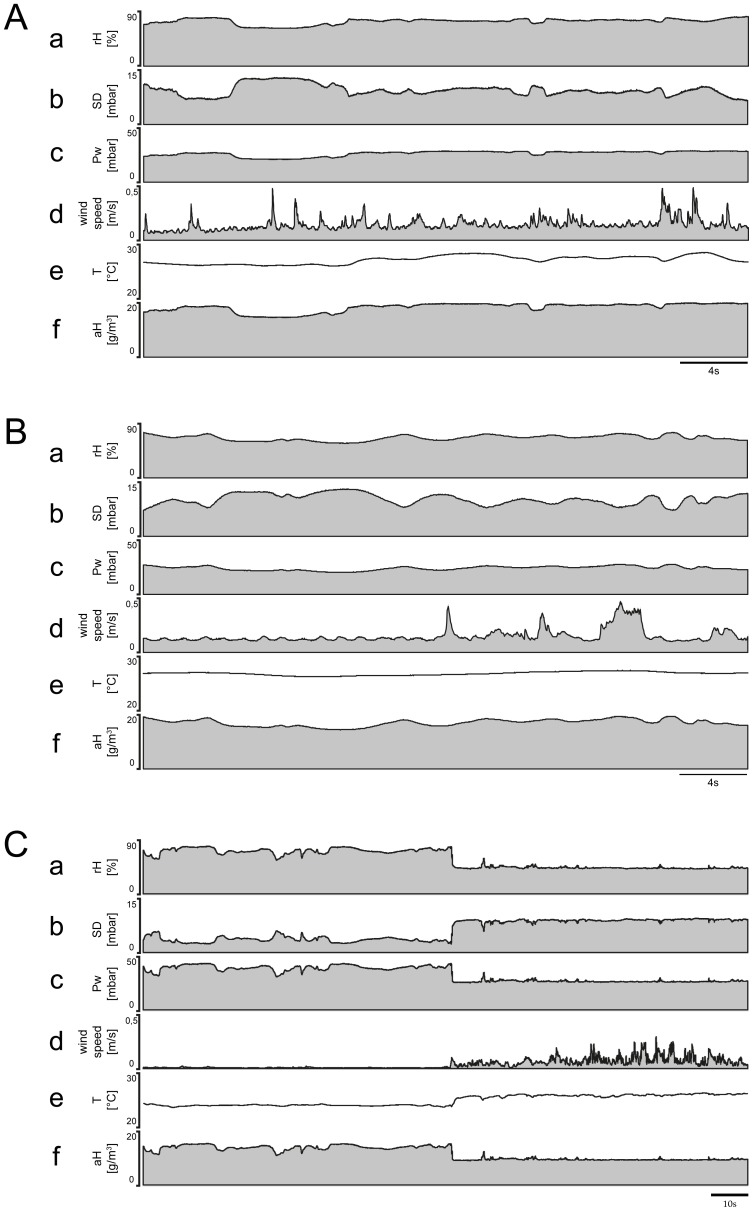
Temporal profiles of absolute humidity, temperature and wind speed recorded while traveling through different habitats. **A.** Open grassy field. **B.** Deciduous forest. **C.** Edge habitat characterized by overhanging canopy and grassland. In each habitat, temporal profiles of relative humidity (*a*), saturation deficit (*b*) and water vapor pressure (*c*) were determined from the measured values of absolute humidity (*f*) and air temperature (*e*). Peak wind speeds (*d*) were not reflected in the profiles of relative humidity (*a*), saturation deficit (*b*) and water vapor pressure. *aH* absolute humidity, *Pw* water vapor pressure, *rH* relative humidity, *SD* saturation deficit, *T* temperature.

These experiments center on the questions of what kind of humidity stimuli a honey bee may encounter during long-distance flights. We made 25 measurement trips using an automobile that traveled along vegetated roadsides, passing (*i*) open farm fields and areas of short grass with clusters of trees shadowing the ground (Fig. 8*A*), (*ii*) through deciduous forests with low canopy cover (Fig. 8*B*), and (*iii*) through fragmented forests with transitions (edges) to grasslands with ruderals and other herbs (Fig. 8*C*). The habitat type significantly affected the humidity fluctuations, with the strength of the effect varying with the recorded absolute humidity (Figs. 8*A* *f, Bf, Cf*). Habitat affected the extremes of humidity peaks and troughs as well as the mean values. Specifically, the humidity transitions were more round and smooth in open grassy fields (Fig. 8*A* *a,b,c*) than in deciduous forests (Fig. 8*B* *a.b.c*), but they were abrupt in edge habitats (Fig. 8*C* *a,b,c*). In open grassy fields, light winds were interspersed with brief periods during which wind speed fluctuated or remained broadly stable at a new level (Fig. 8*A* *d*). Air temperature was relatively constant (Fig. 8*A* *e*). In fragmented deciduous forests, however, rapid changes in wind speed were evident, and the rate at which peaks occurred ranged considerably (Fig. 8*B* *d*). Temperature tended to fluctuate constantly rather than peak (Fig. 8*B* *e*). Crossing the edges of a fragmented habitat yielded rapid changes in wind speed with multiple peaks (Fig. 8*C* *d*). In the same situation, temperature changed quickly and fluctuated around a new value (Fig. 8*C* *e*). A comparison of the temporal profiles of the parameters measured in different landscapes revealed that the absolute humidity (Fig. 8*A–C* *c,f*) affects the relative humidity (Fig. 8*A–C* *a*) and the saturation deficit (Fig. 8*A* *–*–*Cb*) more than it does the temperature (Fig. 8*A–C* *e*) or wind speed (Fig. 8*A–C* *d*).

**Figure 9:**
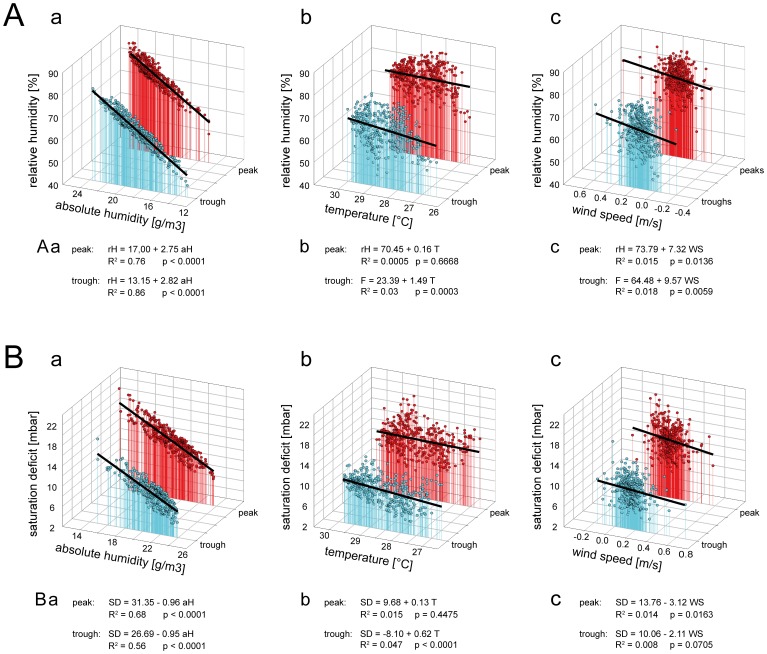
Relationships between humidity fluctuation values, temperature and wind speed. **A**. Peak and trough values of relative humidity plotted as functions of absolute humidity (*a*), temperature (*b*) and wind speed (*c*). **B**. Peak and trough values of saturation deficit plotted as functions of absolute humidity (*a*), temperature (*b*) and wind speed. Functions approximated by linear regressions. *aH* absolute humidity, *R^2^* coefficient for determination, *rH* relative humidity, *SD* saturation deficit, *T* temperature, *WS* wind speed.

The temporal profiles in Fig. 8*A–C* also clearly show that humidity changed mostly smoothly rather than abruptly from one direction to another. The general character of the three measurement trips is the same as has been observed repeatedly in similar habitats. Twelve of the 25 measurement trips were analyzed; each lasted an average of 96 min and covered distances of approximately 25 km. Statistical values of two representative measurement trips are shown in Tab. 2 and 3. In the open grassy field with clusters of trees, the relative humidity fluctuated on average between 38.9 and 33.6%; the maximum amplitude was 13.2%, the mean amplitude 5.3%. The maximum upward and downward rates of humidity ranged from –9.4 to +12.3%/s, the mean upward and downward rates of humidity change from +1.4 to –1.3% rH/s (Tab. 2). The rate with which wind speed changed ranged from –1.2 to +1.4 m/s^2^, the rate of temperature change from –0.03 to +0.02°C/s. Thus, for a honey bee flying at a mean speed of 7.4 m/s, the peaks and troughs of the relative humidity occurred at a mean interval of 8.0 s, the peaks and troughs of the temperature at a mean interval of 66.6 s and the peaks and troughs of the wind speed at a mean interval of 6.6 s. Hence the mean distance between the peaks and troughs of the relative humidity was 62.2 m, between those of the temperature 518.5 m, and between those of the wind speed 51.9 m. In the fragmented deciduous forest with low canopy cover, the relative humidity fluctuated on average between 62.5 and 76.9%, the maximum amplitude was 25.7% and the mean amplitude was 14.2%. The maximum upward and downward rates of humidity ranged from –13.5 to +26.8%/s, the mean upward and downward rates of humidity change between –2.6 and +2.8% *rH*/s (Tab. 3). The rate with which the wind speed changed ranged from –0.3 to +0.3 m/s^2^, the rate of temperature change from –0.1 to +0.1°C/s. Thus, for a honey bee flying at a mean speed of 7.7 m/s, the peaks and troughs of the relative humidity occurred at a mean interval of 8.9 s, those of the temperature at a mean interval of 8.8 s and those of wind speed at a mean interval of 10.9 s. Hence the mean distance between the peaks and troughs of the relative humidity was 59.7 m, between those of the temperature was 59.3 m, and those of the wind speed 72.8 m. Overall, the statistical values obtained from the two habitats indicate differences in both maximum and mean values of the various humidity parameters and, as a consequence, in the rate with which humidity changes. Periods between temperature changes were fewer in the open grassy field than in the deciduous forest, resulting in higher rates of temperature change in the latter. Concerning wind speed changes, the periods were longer and the rates of change smaller in the deciduous forest versus grassy field. The observed fluctuating changes in the relative humidity or the saturation deficit may be the result of various entraining processes caused by turbulent air, radiation and topographic variables such as slope and aspect. For the 12 measurement trips, we determined the dependence of the peaks and troughs of relative humidity and the saturation deficit on the absolute humidity, the temperature and the wind speed. Fig. 9 gives an example of the fragmented deciduous forest (Tab. 3), whose various humidity parameters show greater variability than the open grassy field (Tab. 2). The peaks and troughs of both the relative humidity and the saturation deficit are strongly dependent on the absolute humidity (Fig. 9*A* *a,Ba*). Moreover, when the absolute humidity is high, the peaks and the troughs are also high, and when the absolute humidity is low, the peaks and troughs are also low. The almost parallel slopes of the regression lines used to approximate the dependences of the peaks and troughs of the relative humidity and the saturation deficit on the absolute humidity indicate that the fluctuation amplitudes are almost the same at different absolute humidities. By contrast to the absolute humidity, the peak and trough of the relative humidity were affected neither by temperature (Fig. 9*A* *b,Bb*) nor wind speed (Fig. 9*A* *c,Bc*). Similarly, the peak values of the saturation deficit display no dependence on temperature or wind speed. However, the trough values of the saturation deficit slightly decrease with increasing temperature.

**Table 2:** Fluctuation statistics of humidity, temperature and wind speed values for a typical measurement trip through an open grassy field.

Measurement trip	open grassy field			
Trip duration	90 min			
Trip distance	42 km			
Mean travel speed	7.4 m/s			
**Parameters**	**relative humidity**	**saturation deficit**	**temperature**	**wind speed**
Number of peaks	329	217	41	405
Number of troughs	328	216	40	404
Number of transients	675	433	81	809
Mean interval between transients	8.0 s	12.4 s	66.6 s	6.6 s
Mean distance between transients	62.2 m	96.9 m	518.5 m	51.9 m
Maximum peak value	45.6	37.6	33.8	+3.1
Minimum trough value	27.3	28.4	32.8	−2.2
unit	[%]	[mbar]	[°C]	[m/s]
**Maximum amplitude**	**13.2**	**6.8**	**0.5**	**2.9**
Minimum amplitude	3.0	2.0	0.1	0.9
unit	[%]	[mbar]	[°C]	[m/s]
Mean peak value	38.9±2.7	34.6±1.2	33.6±0.1	+1.35±0.36
Mean trough value	33.6±3.6	31.2±1.2	33.3±0.1	−1.06±0.22
**Mean amplitude**	**5.3±1.8**	**3.3±1.0**	**0.3±0.08**	**1.2±0.2**
unit	[%]	[mbar]	[°C]	[m/s]
Maximum rate of change				
upward	+12.3	+4.3	+0.1	+34.8
downward	−9.4	−4.7	−0.1	−41.6
unit	[% rH/s]	[mbar/s]	[°C/s]	[m/s^2^]
Mean rate of change				
upward	+1.3±1.1	+0.5±0.4	+0.02±0.02	+1.4±3.0
downward	−1.4±1.1	−0.6±0.5	−0.03±0.02	−1.2±2.9
unit	[% rH/s]	[mbar/s]	[°C/s]	[m/s^2^]

The mean peak values, the mean trough values, the mean amplitudes between peaks and troughs, as well as the mean rates of upward change and of downward change are significantly different (t-test, *p*<0.001) from the corresponding values of the deciduous forest (Table 3).

**Table 3:** Fluctuation statistics of humidity, temperature and wind speed values for a typical measurement trip through a deciduous forest.

Measurement trip	fragmented deciduous forest			
Trip duration	120 min			
Trip distance	48 km			
Mean travel speed	7.7 m/s			
**Parameters**	**relative humidity**	**saturation deficit**	**temperature**	**wind speed**
Number of peaks	402	398	405	330
Number of troughs	401	397	404	329
Number of transients	803	795	809	659
Mean interval between transients	8.9 s	9.0 s	8.8 s	10.9 s
Mean distance between transients	59.7 m	60.3 m	59.3 m	72.8 m
Maximum peak value	87.5	21.6	30.0	+1.7
Minimum trough value	45.2	4.5	26.4	−0.4
unit	[%]	[mbar]	[°C]	[m/s]
**Maximum amplitude**	**25.7**	**19.9**	**1.8**	**1.6**
Minimum amplitude	4.9	1.9	0.3	0.5
unit	[%]	[mbar]	[°C]	[m/s]
Mean peak value	76.9±5.4	13.8±2.5	29.0±0.6	+0.5±0.2
Mean trough value	62.5±6.4	9.4±2.1	28.4±0.7	−0.1±0.1
**Mean amplitude**	**14.2±3.7**	**4.4±1.3**	**0.6±0.2**	**0.6±0.1**
unit	[%]	[mbar]	[°C]	[m/s]
Maximum rate of change				
upward	+26.8	+19.7	+1.0	+7.5
downward	−13.5	−7.8	−1.1	−3.6
unit	[% rH/s]	[mbar/s]	[°C/s]	[m/s^2^]
Mean rate of change				
upward	+2.8±2.4	+1.5±1.0	+0.1±0.1	+0.3±0.7
downward	−2.6±2.9	−1.2±1.0	−0.1±0.1	−0.3±0.5
unit	[% rH/s]	[mbar/s]	[°C/s]	[m/s^2^]

The mean peak values, the mean trough values, the mean amplitudes between peaks and troughs, as well as the mean rates of upward change and of downward change are significantly different (t-test, *p*<0.001) from the corresponding values of the open grassy field (Table 2).

## Discussion

### Humidity stimulation and the question of the adequate stimulus

In his study of the cockroach's hygroreceptors, Yokohari (1978) suggested that hygroreceptors function as mechanical hygrometers. He provided a descriptive model based on the assumption that the cuticular wall of the sensillum acts as a hygro-mechanical transducer that changes its dimension due to the uptake (adsorption) and loss (desorption) of water like a hair hygrometer. This water uptake detector functions because a human or horse hair under tension becomes longer when the relative humidity rises and shorter when it falls. It became accepted wisdom that relative humidity is the adequate stimulus. However, the relative humidity is not a direct measure of the water vapor in the air. Identical relative humidity values do not indicate identical atmospheric moisture conditions unless the temperature is also the same. A 50% relative humidity at low temperatures contains much less water vapor than a 50% relative humidity at high temperatures. The reason is that the higher the temperature, the more thermal energy is in the air and the more evaporative work can be done in that air parcel. Evaporation will therefore occur more rapidly from a moist surface (under the same conditions) into an air with a relative humidity of 50% at high than at low temperature. The relative humidity of the air alone does not indicate the moisture conditions of the air. An air parcel with a 50% relative humidity is dry when the temperature is high but wet when the temperature is low: 50% relative humidity indicates dryness at high temperatures or wetness at low temperatures. Relative humidity is a useful measure of evaporation provided that moisture conditions are being assessed at a single constant temperature. Thus if an insect defends its water content by keeping evaporation constant, it must maintain a constant vapor pressure deficit and search for regions of similar saturation deficit. To maintain the same saturation deficit at high temperatures, the relative humidity must be increased and vice versa. If observations are to be extended over a range of temperatures, however, the saturation deficit is the exact measure. For this reason we recorded the water vapor pressure, the saturation deficit as well as the relative humidity. However, nothing is known about the amplitudes and rates of both humidity and temperature changes encountered by a running or flying insect in its natural environment. In this study we addressed these issues by mobile measurements of the fluctuating strength of humidity and temperature in different landscapes.

### Mobile humidity measurements

Mobile humidity measurements from an automobile have the disadvantage of being confined to passable landscapes. Nonetheless, the measurements can be made representative very easily by repeating a trip so that humidity values at different times or weather conditions can be quantified for given landscapes; the common evaluation method facilitates data interpretation. Importantly, this study did not attempt to characterize the microclimatic conditions of various landscapes. The potential effects of landscape structures such as topography, slope and aspect, or composition, density and heights of vegetation were not considered. Moreover, the measurements were done on calm and clear summer days, which are no doubt the most suitable for bee flight activity.

The important finding was that the dominant form of changes in the absolute humidity was slow, regardless of landscape type. Accordingly, the rates of changes in relative humidity and the saturation deficit were low. The landscapes, however, differ in the range and amplitude of humidity changes. In contrast, the temperature changes were small in the different landscapes; similarly small was their effect on the humidity peak and trough values, regardless of how humidity was expressed. Wind speed generally changed more pronouncedly than temperature but without affecting the humidity peaks or troughs.

### Hygroreceptor sensitivity

The first recordings from a single sensillum containing a hygroreceptor were reported by Lacher (1964) 50 years ago. He identified a moist cell on the antenna of the worker bee. Waldow (1970) demonstrated electrophysiologically on the antenna of the locust a dry cell together with a moist cell in the same sensillum. Yokohari et al. (1983) reinvestigated the worker-bee's hygroreceptive sensilla and found a moist cell and a dry cell combined in a single sensillum with a cold cell. Stimulation consisted of rapid, step-like changes in relative humidity. To this end, the moist cell was adapted to extremely dry air (0% *rH*) and then stimulated with higher humidity levels up to saturated air. Conversely, the dry cell was adapted to extremely moist air (100% *rH*) and then stimulated with lower humidity levels down to dry air. In the moist cell, impulse frequency was higher the higher the humidity level used for stimulation, and conversely, the dry-cell's impulse frequency was higher the lower the stimulating humidity level. Nonetheless, the technique used to stimulate the worker-bee's hygroreceptors requires comment [12]. The significant stimulus parameter could be the rate with which the humidity stimulus arrived at the sensillum rather than the humidity level. Unfortunately the rate of humidity change is almost impossible to accurately determine for rapid changes because the time course of the humidity recorded during the change is that of the hygrometer, not of the sensillum. Even if the velocity of the humidity change is constant in a series with different humidity levels, the rate of humidity change will be faster the larger the difference between adaptation humidity and the humidity level used for stimulation. Despite these difficulties, the study of the worker-bee's hygroreceptors showed that the greater the humidity change (more precisely, the greater the amplitude and the rate of change), the stronger the response of the moist and dry cells [12]. This basic relationship is also found in the drone's hygroreceptors: the greater the amplitude of humidity oscillations, the stronger the response of both cells.

**Figure 10:**
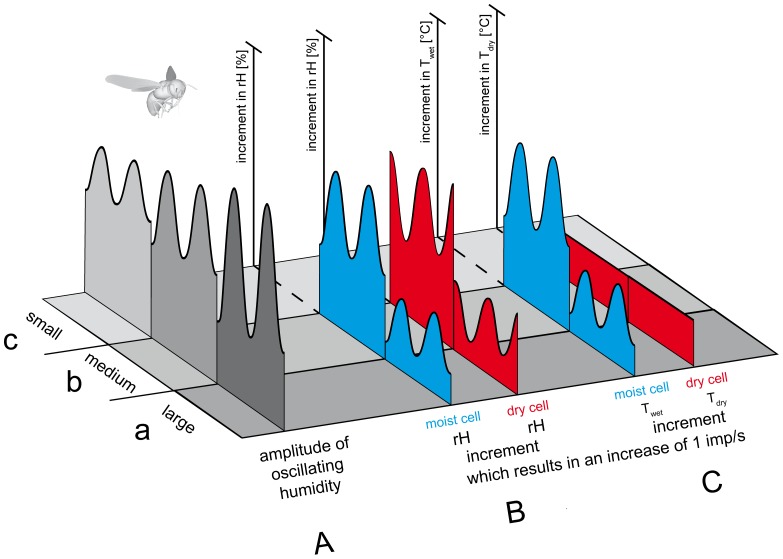
Sensitivity of a honeybee's moist cell and dry cell for oscillating humidity changes with different amplitudes. Sensitivity is defined as the reciprocal of the stimulus increment regarded to produce a neural response of unit size (1 imp/s). **A.** Amplitude of humidity oscillations. *a* small amplitude oscillations, *b* medium amplitude oscillations, *c* large amplitude oscillations. **B**. Sensitivity of moist and dry cells that measures the relative humidity of the air. *a* During small amplitude humidity oscillations the increments in relative humidity required for unit change in the discharge rates of the moist and dry cells are large. *b* During medium amplitude humidity oscillations, somewhat smaller increments in relative humidity are required for unit change in the discharge rates of the moist and dry cells. *c* During large amplitude humidity oscillations, small increments in relative humidity may elicit the same unit change in the discharge rates of both cells.

The range over which humidity oscillated determined the response magnitude of the moist and dry cells as well as their sensitivity. This magnitude decreased with diminishing oscillation amplitude. In effect, they responded well to large and poorly to small amplitude oscillations. In accordance with the responsiveness, sensitivity decreased with diminishing oscillation amplitude. Sensitivity is usually described by the reciprocals of the slope values of the input-output functions, i.e., the increment in stimulus strength required to change impulse frequency by 1 imp/s. Fig. 10 visualizes the effect of the stimulus amplitude on sensitivity. At large-amplitude humidity oscillations (∼50% *rH*), a 1 imp/s increase in the moist-cell's discharge can be elicited by increasing instantaneous humidity by +3.3%. In the same situation, it takes a –2.5% decrease to increase frequency of the dry cell by 1 imp/s. When the oscillation amplitude is reduced to a medium value (∼30% *rH*), it takes in the moist cell a +5% increase in instantaneous humidity for a 1 imp/s increase; in the dry cell the value is –5%. A further reduction of the oscillation amplitude to a small value (∼10% *rH*) requires even larger humidity changes to affect the discharge rates of the two cells by the same amount.

### What determines sensitivity?

Insects are believed to regulate their water content by avoiding areas with extremes and by moving along the humidity gradient into a more suitable environment. While avoidance or search behavior is certainly very efficient in dealing with the insect's water balance, it has the disadvantage that the insect's entire activity is devoted to improving its humidity environment. It therefore competes with other forms of behavior and its presence at a given time means that humidity is at that time the most important motivation for the insect. In trying to understand the contribution of hygroreceptors to specific behaviors, the responses of the moist and dry cells were plotted as functions of the instantaneous humidity and its rate of change. The range of impulse frequency spanned by the regression planes and their slopes indicate hygroreceptor sensitivity. High values are much more likely to lead to behavioral responses than low ones. Reduction in sensitivity suggests, but does not strictly demonstrate, a decrease in information content.

In the honeybee's hygroreceptors, high sensitivity is devoted to large-amplitude humidity oscillations, which may seriously affect body water content. Small-amplitude oscillations are associated with low sensitivity, probably because they are less harmful. Accordingly, sensitivity is related not only to the hygroreceptors' inputs but also to the outputs relevant for the specific behavior of improving water balance. Low-amplitude oscillations will be relevant too if they occur in the range of extreme humidities but they may be of little use for orientation as the stimulus field has no definite directions. However, some insect tolerate extremes of their water content and finally carry out escape movements intended to improve the humidity environment.
