# Correction: Sensitivity of Honeybee Hygroreceptors to Slow Humidity Changes and Temporal Humidity Variation Detected in High Resolution by Mobile Measurements

**DOI:** 10.1371/journal.pone.0106878

**Published:** 2014-08-21

**Authors:** The PLOS ONE Staff

The grant number in the funding statement is incorrect. Please refer to the correct funding statement below:

This work was supported by a grant from the Austrian Science Fund (Project P20.196-B17) (www.fwf.ac.at <http://www.fwf.ac.at>). The funders had no role in study design, data collection and analysis, decision to publish, or preparation of the manuscript.
